# Bringing Giftedness to Bear: Generativity, Meaningfulness, and Self-Control as Resources for a Happy Life Among Gifted Adults

**DOI:** 10.3389/fpsyg.2019.01972

**Published:** 2019-09-13

**Authors:** Bernadette Vötter, Tatjana Schnell

**Affiliations:** ^1^Institute of Psychology, University of Innsbruck, Innsbruck, Austria; ^2^MF Norwegian School of Theology, Religion and Society, Oslo, Norway

**Keywords:** giftedness, intellectually gifted, high academic achievement, generativity, meaningfulness, self-control, subjective well-being, moderated mediation analysis

## Abstract

Meaning in life has been found to be of particular importance for the subjective well-being of intellectually gifted individuals. However, there is a lack of research about what contributes to gifted adults’ meaning in life and how it could be enhanced. This study examined if the devotion of one’s gift or talent to the well-being of others—i.e., the source of meaning “generativity”—would lead to a sense of meaning and, in further consequence, result in higher subjective well-being over time. Furthermore, we hypothesized that the effect of meaningfulness on subjective well-being was conditional on trait self-control. Longitudinal data of two gifted groups was obtained via an online study: 100 intellectually gifted individuals (55% female; mean age 43 ± 9 years) and 52 high academic achievers (29% female; mean age 57 ± 14 years). The former group experienced significantly lower levels of meaningfulness (*p* = 0.001, η^2^ = 0.076), self-control (*p* < 0.001, η^2^ = 0.090), and generativity (*p* = 0.025, η^2^ = 0.034) than the latter. As expected, the actualization of generative orientations in life enhanced both gifted groups’ meaningfulness and, in further consequence, their subjective well-being over time. Furthermore, the positive association between life meaning and subjective well-being was enhanced by trait self-control among the intellectually gifted but not among the high academic achievers. However, as proposed, the latter’s subjective well-being was strongly related to self-control. Results highlight that a generative orientation can help gifted individuals to advance a personal sense of meaning and happiness over time. In this context, intellectually gifted individuals appear to particularly benefit from self-control. Consequently, the intrinsic willpower to subdue inner responses, emotions as well as undesired behaviors might strengthen the positive effect between sources of meaning, life meaning, and subjective well-being.

## Introduction

Meaning in life is gaining increased attention among empirical scholars as well as in society (cf. Antonovsky, 1979; Seligman and Csikszentmihalyi, 2000; Schnell, 2016; Yeoman et al., 2019). Its psychological roots go way back to the founder of logotherapy Viktor E. Frankl (Frankl, 1985). In his pioneering work, he proposed that the search for meaning is a primary force in humans’ lives (e.g., Frankl, 1985, 1996). Instead of searching for meaning, however, it is the experience of meaningfulness and crisis of meaning that have repeatedly been shown to impact human lives. Meaningfulness is based on an experience of one’s life as coherent, significant, directed, and belonging (Schnell, 2009). Coherence originates when life is evaluated as comprehensible and consistent throughout various life domains. A sense of significance emerges when one’s actions resonate, are perceived by others, or produce results. Direction refers to a meta-orientation throughout life, guiding decisions, choices and goal pursuit. Belonging refers to a sense of having a place in this world, of being part of a bigger entity, e.g., humanity, society, family, or confession. Various empirical studies have emphasized the importance of meaning in life for humans’ psychological well-being, optimism, life satisfaction, self-efficacy, as well as physical and mental health (e.g., Ryff, 1989; King et al., 2006; Park, 2010; Roepke et al., 2013; Czekierda et al., 2017; Pollet and Schnell, 2017; Vötter and Schnell, 2019a). Individuals with a high level of meaning in life reported more happiness, lower depression, anxiety, and stress (e.g., Zika and Chamberlain, 1992; Debats, 1996; Mascaro and Rosen, 2005; Schnell, 2009; Damásio et al., 2013). Meaning in life has also been found to be positively associated with age (Steger et al., 2006; Schnell and Becker, 2007; Pedersen et al., 2018).

An absence of meaningfulness can be met with indifference, as in existential indifference (Schnell, 2010; Damásio and Koller, 2015). It is only when an individual actually misses a sense of meaningfulness and suffers from this lack of meaning in life, a crisis of meaning occurs (Schnell, 2009). Crises of meaning are states of deep suffering, often paired with anxiety, depression, pessimism, and negative affect (Schnell, 2016). They are negatively related to resources such as self-efficacy, resilience, self-regulation, or religiosity (Schnell, 2009; Damásio et al., 2013; Hanfstingl, 2013; Routledge et al., 2017; Pedersen et al., 2018; Sørensen et al., 2019). Moreover, crises of meaning have been established as a major risk factor for suicide (Schnell et al., 2018).

Meaning in life can be derived from various sources of meaning (e.g., Battista and Almond, 1973; O’Connor and Chamberlain, 1996; Wong, 1998; Debats, 1999; Schnell, 2009). Sources of meaning are characterized as basic orientations “underlying human cognition, behavior and emotion” (Schnell, 2009, p. 486). They promote commitment, structure, and direction toward different dimensions of life, which in further consequence are proposed to lead to a sense of meaning (e.g., Leontiev, 1982; Schnell, 2009). Among the sources of meaning, generativity is the best predictor of meaning in life (e.g., Emmons, 2005; Schnell, 2011; Damásio and Koller, 2015; Pedersen et al., 2018). As part of his life span theory, Erikson defined generativity as “primarily the concern in establishing and guiding the next generation” (Erikson, 1963, p. 276). He argued that generativity is a significant developmental task to accomplish during middle adulthood (from 30 to 65 years). Individuals who fail to be generative are likely to experience a sense of stagnation and have a higher risk of poor psychosocial adjustment in midlife (Erikson, 1963). Generativity has been positively associated with psychological well-being (Sheldon and Kasser, 2001; Grossbaum and Bates, 2002; An and Cooney, 2006; Rothrauff and Cooney, 2008), mental health (McAdams and de St. Aubin, 1992; McAdams and Logan, 2004), and meaning in life (Schnell, 2011; Hofer et al., 2014). Studies have shown that individuals with higher levels of generativity reported higher life satisfaction, lower levels of anxiety, depression, and neuroticism as well as a lower risk of mortality (McAdams et al., 1998; Ackerman et al., 2000; McAdams and Logan, 2004). Furthermore, generative goals have been positively associated with age (e.g., McAdams et al., 1993; Sheldon and Kasser, 2001). Thus, the older an individual, the more important generative goals become. Various ways lead to generativity, including passing on one’s knowledge and experiences to others, parenthood, volunteering, mentoring, teaching, philanthropy, nursing, institutional involvement, and civic engagement (e.g., Rossi, 2001; Schnell and Hoof, 2012).

### Intelligence and Meaning in Life

Even though human intelligence is one of the most investigated factors in the field of psychology, and the concept of giftedness is widely used, no commonly accepted definition of giftedness exists yet. However, the literature offers two main criteria to narrow down the heterogeneous construct of giftedness. The first criterion is high general intelligence, which can be measured by the quotient of intelligence (IQ; mean = 100, standard deviation = 15) established by an intelligence test (e.g., Spearman, 1904; Bortz, 2005). Thus, intellectual giftedness is attributed to individuals who reach IQ scores of at least 130 (e.g., Wirthwein and Rost, 2011; Preckel and Vock, 2013), which indicates that the individual has higher cognitive abilities than 98% of the population (e.g., Bortz, 2005; Rost, 2013). The second criterion to classify a person as gifted is superior (academic) achievement or performance (e.g., Ziegler and Raul, 2000; Wirthwein and Rost, 2011; Preckel and Vock, 2013; Rost, 2013).

High cognitive abilities, as well as other exceptional talents, can be viewed as ambivalent gifts. They carry huge potential on the one hand, but are often linked with stigmatization, on the other (e.g., Baudson and Ziemes, 2016). Therefore, gifted individuals’ life-worlds are bound to differ from the general population’s (Coleman, 2012). In spite of that, very little is known about gifted individuals in their adulthood. For the main part, research has focused on needs of gifted children (e.g., Kennedy, 1995; Coleman and Cross, 2000; Tieso, 2007; Cross, 2011; Lamont, 2012). But how do they fare when they grow up? Only very few studies have tackled this question (e.g., Lubinski et al., 2006; Wirthwein and Rost, 2011; Dijkstra et al., 2012; Pollet and Schnell, 2017; Karprinski et al., 2018; Vötter and Schnell, 2019a, b).

A recent cross-sectional study by Vötter and Schnell (2019b) examined life meaning and subjective well-being among gifted adults. They found intellectually gifted adults at a higher risk of suffering from a crisis of meaning than high academic achievers and a control group. The intellectually gifted also experienced lower meaningfulness as well as subjective well-being than a control group with an average IQ (see also Pollet and Schnell, 2017). Considering the four pillars of meaning, i.e., coherence, significance, orientation, and belonging (Schnell, 2009, 2014), the gifted might find it particularly challenging to experience a sense of *belonging*. A strong sense of being “different”, a “misfit”, was often reported in interviews conducted with gifted adults who were characterized by low meaningfulness (Wittmann, 2019). Roeper (1991) described gifted adults as feeling fundamentally different from others. Also Stålnacke and Smedler (2011), who studied adult members of the Swedish Mensa Society, came across such experiences of being different. *Coherence* could be another weakness in the lives of gifted adults. This is exemplified by a lack of fit between personal interests and competences and professional position. As reported by Pollet and Schnell (2017), intellectually gifted adults experienced significantly lower meaning as well as joy in their workplace. A study on intellectually gifted individuals’ work satisfaction found that most participants accepted their situation at work, albeit rather indifferently (Persson, 2009). In additional interviews, being ignored or misunderstood by employers were mentioned as major reasons for this attitude. Finally, experiencing a sense of *significance* might be taxing for intellectually gifted adults. Giftedness is typically viewed as a talent that should be multiplied and brought to fruition. Such expectations are early internalized by the gifted, often accompanied by a pressure to perform in order to maintain their extraordinary status (Mofield and Parker Peters, 2018) and these exaggerated expectations might have a paralyzing effect.

In contrast to the intellectually gifted, superior academic achievers reported similar levels of meaningfulness, crisis of meaning, and subjective well-being to the mentioned control group (Vötter and Schnell, 2019b). These results also suggest a specific link between high intelligence and issues related to finding meaning in life.

### The Present Study

Longitudinal findings by Vötter and Schnell (2019a) established a sense of meaning in life as a crucial predictor for intellectually gifted adults’ subjective well-being, operationalized as satisfaction with life, the presence of positive emotions (e.g., euphoria, optimism, happiness, or love) as well as the absence of negative emotions (e.g., anger, anxiety, or grief) (e.g., Ryan and Deci, 2001; Emmons, 2003; Kim-Prieto et al., 2005; Fredrickson, 2009). This underlines the importance of strengthening gifted individuals’ meaning in life in order to increase their chances of leading a happier life. But what contributes to gifted adults’ life meaning, and how could it be enhanced? Since generativity has repeatedly been established as an outstanding predictor for meaning in life (e.g., Frankl, 1996; Emmons, 2005; Schnell, 2011) we propose that intellectually gifted adults, due to their higher risk of suffering from an existential crisis and experiencing lower meaningfulness in life (Vötter and Schnell, 2019b), might particularly benefit from a generative orientation in life. Such an orientation typically results in actions that are experienced as significant and, by contributing to a greater good, create a sense of belonging. Moreover, actions performed for the good of others have been found to be positively associated with well-being (Niemiec et al., 2009; Martela and Ryan, 2016). Thus, we hypothesized that the devotion of one’s gift or talent to the well-being of others will lead to a sense of meaning in life among (a) intellectually gifted adults, as well as (b) high academic achievers, and that this experienced meaningfulness will, in further consequence, result in higher subjective well-being over time (hypotheses 1a,b). Considering recent findings of a tendency for psycho-social maladjustment among the intellectually gifted (Karprinski et al., 2018; Vötter and Schnell, 2019b) we expected generativity and meaningfulness to be higher in the high academic achievers group (hypothesis 2).

According to De Ridder et al. (2012), high academic achievement is closely associated with self-control. Self-control is defined as the ability to override or modify one’s inner responses as well as to interrupt undesired behaviors (Tangney et al., 2004; Vohs and Baumeister, 2004). Self-control has been positively associated with happiness (Cheung et al., 2014), life satisfaction (Hofmann et al., 2014), self-esteem (Tangney et al., 2004), a more effective inhibition of negative emotional response (Kieras et al., 2005), increased motivation (Muraven and Slessareva, 2003), and academic success (Tangney et al., 2004; Duckworth and Seligman, 2005). Furthermore, individuals with higher levels of self-control reported less depression and anxiety (Bowlin and Baer, 2012). Findings by Moffitt et al. (2011) suggest that self-control can predict health and well-being outcomes up to 30 years later. Taking these associations into account, we proposed that self-control might be a personality factor in which the two gifted populations differ, with particularly high values to be found among high academic achievers (hypothesis 3). Moreover, we hypothesized that the positive effect of meaningfulness on subjective well-being might be conditional on trait self-control in both gifted groups (hypotheses 4a,b). Experiences of meaning ensue from purpose put into action (Schnell, 2014, 2020). They are based on an agentic and involved life-style that corresponds to personal values. Individuals with a high level of self-control are known to be able to inhibit undesired behaviors and promote desirable action. This provides a good basis for living one’s life in a self-determined way, pursuing goals that are in line with personal sources of meaning and thus finding fulfillment and—probably as a byproduct (Schnell, 2013, 2014)—subjective well-being.

Examining the impact of generativity on subjective well-being via a sense of meaning as well as the moderating role of self-control among varying gifted groups might help to determine why some gifted adults are able to live a happy life while others suffer from an existential crisis. The proposed moderated mediation model was tested with longitudinal data from 152 gifted adults, separated in two groups: intellectually gifted (*N* = 100) and high academic achievers (*N* = 52).

## Materials and Methods

### Procedure and Sample

Longitudinal data was obtained from 152 gifted adults via an online survey (employing the survey tool limesurvey) at two times of measurement (t1, t2), as part of a larger research project. The questionnaire included socio-demographic variables (age, gender, education, and family status) and four scales to assess generativity (t1), meaningfulness (t1), subjective well-being (t2), and self-control (t2). Participants had to answer all items by default. Thus, no missing data occurred. Only fully completed questionnaires were included in the analyses. Considering the aforementioned two criteria to classify giftedness (e.g., Wirthwein and Rost, 2011; Preckel and Vock, 2013) and the recommendation of several scholars to distinguish between giftedness as potential and giftedness as high performance (Wirthwein, 2010; Pollet and Schnell, 2017), we recruited two groups: one matched the criterion of high general intelligence, the other matched the criterion of high academic achievement.

The first group—the intellectually gifted—was labeled as high IQ group (HIQ). They were recruited through the German and Austrian high IQ society Mensa. The requirement for membership is an IQ of at least 130, measured after supervised administration of an approved intelligence test. Thus, all members meet the criterion to be classified as intellectually gifted (e.g., Preckel and Vock, 2013). To reach out to as many Mensa members as possible, we contacted the Austrian and German branch representatives of Mensa and asked them to forward an invitation to their members. This included a short description of the study and a link to the online-survey. At time 1, 148 Mensa members participated. Among them, 102 gave approval to be contacted for a follow-up survey. When we contacted them 4 years later via e-mail, 100 agreed to participate in the follow-up study. Accordingly, there was an attrition rate of 32% between the two times of measurement. An ANOVA showed no significant differences regarding age (*F*(1,146) = 0.218, *p* = 0.642), gender (*F*(1,146) = 3.106, *p* = 0.080), family status (*F*(1,146) = 0.078, *p* = 0.781), meaningfulness (*F*(1,146) = 0.419, *p* = 0.518), and generativity (*F*(1,146) = 1.057, *p* = 0.306) among those who participated at both times of measurement and those 48 participants who dropped out after the first time of measurement. Consequently, no attrition bias was anticipated for the longitudinal analyses. A *post hoc* power analysis (G^∗^Power; Faul et al., 2007) was conducted to determine the power of the study based on the sample size of 100 and the determination of at least a medium effect size (*f*^2^ = 0.15, α = 0.05). Results indicated adequate statistical power (1−β = 0.99) for the intellectually gifted sample. All subsequent analyses were conducted on the data of those 100 participants who participated at both times of measurement. Of these, 55% were female, with a mean age of 43 ± 9 years at t2. Most were Germans (83%), followed by 16% Austrians, and 1% of other nationalities. Fifty-one percent of the participants were married, 25% were single, 22% were in a relationship, and 2% were divorced. Thirteen percent were holders of a doctoral degree, 62% had graduated from university, 19% were high school graduates, 4% had completed an apprenticeship or vocational secondary school, and 2% had completed general education.

The second group was labeled as high academic achievers (HAA). For recruitment reasons, we contacted 724 Austrian academic award winners who obtained their doctorate *sub auspiciis praesidentis rei publicae*. This academic award is the highest possible distinction up to Ph.D. level in Austria. From 1952 until 2012, 1042 individuals received this academic honor; only 29% of them were female (Grabenweger, 2012). A candidate qualifies for this award, which is handed over by the Austrian Federal President, if all grades from high school up to tertiary education are excellent, and if the candidate graduated with highest honors (summa cum laude) at every level. All members of this group thus met the criterion of giftedness via high academic achievement (e.g., Preckel and Vock, 2013). Ninety-two academic award winners participated in the online survey at t1. All of them gave approval to be contacted for a follow-up study. Four years later, when we contacted them again, 52 agreed to participate in the follow-up study. Hence, there was an attrition rate of 43% between the two times of measurement. An ANOVA showed, equally to the results of the intellectually gifted group, no significant differences relating to age (*F*(1,90) = 0.385, *p* = 0.536), gender (*F*(1,90) = 0.000, *p* = 0.985), family status (*F*(1,90) = 0.613, *p* = 0.436), meaningfulness (*F*(1,90) = 0.281, *p* = 0.597), and generativity (*F*(1,90) = 0.076, *p* = 0.783) among those who participated at both times of measurement and those 40 who dropped out after the first time of measurement. No attrition bias was therefore anticipated for the longitudinal analyses. A *post hoc* power analysis (G^∗^Power; Faul et al., 2007) was conducted to determine statistical power based on the sample size of 52 and the determination of at least a medium effect size (*f*^2^ = 0.15, α = 0.05). Results indicated adequate power of the study (1−β = 0.87) also for the high academic achiever sample. All subsequent analyses were conducted on the data of those 52 participants who participated at both times of measurement. Of these, 29% were female, with a mean age of 57 ± 14 years at t2. All of them were Austrians. Six percent were single, 11% were in a relationship, 77% were married, and 6% were divorced. Due to the selection criteria for this group (doctorate *sub auspiciis praesidentis rei publicae)*, this group was over-proportionally well-educated with 100% holders of a doctoral degree.

This study was carried out in accordance with the Declaration of Helsinki. Participation was entirely voluntary. All participants declared their consent by signing up for participation of their own accord, after receiving a written invitation and study information. All participants were made aware that they could withdraw from the study at any time.

### Measures

At t1, generativity was assessed by the six item generativity scale from the Sources of Meaning and Meaning in Life Questionnaire (SoMe; German version: LeBe; Schnell and Becker, 2007; Schnell, 2009). The scale measures the extent of the respondent’s desire to encourage the next generations’ well-being by doing and creating things that outlive the self (e.g., “I strive to do something for future generations”). Items are rated on a 6-point Likert scale ranging from 0 (“strong disagreement”) to 5 (“strong agreement”). Internal consistency in the current study was good (α_HIQ_ = 0.78; α_HAA_ = 0.84).

At t1, meaningfulness was measured by the respective scale from the Sources of Meaning and Meaning in Life Questionnaire (SoMe; German version: LeBe; Schnell and Becker, 2007; Schnell, 2009). The scale assesses the degree of perceived meaningfulness in life (e.g., “I have a task in my life”). The five items are rated on a 6-point Likert scale, with 0 indicating strong disagreement and 5 indicating strong agreement. The meaningfulness scale had a good internal consistency in the present study (α_HIQ_ = 0.78; α_HAA_ = 0.78).

At t2, subjective well-being was assessed by the WHO-5 Wellbeing Index (WHO-5; Brähler et al., 2007). The scale includes five items and was designed to measure subjective well-being, based on positive mood (e.g., “I am feeling cheerful and in good spirits”) and vitality (e.g., “I am feeling energetic and active”). Items are rated on a 6-point Likert scale, with 0 indicating at no time and 5 indicating all the time. WHO-5 demonstrated a good internal consistency in the current study (α_HIQ_ = 0.82; α_HAA_ = 0.80).

At t2, trait self-control was measured using the short German version of the self-control scale (SCS-KD; Tangney et al., 2004; Bertrams and Dickhäuser, 2009). The scale measures the ability to override or modify inner responses and to interrupt undesired behaviors. The 13 items (e.g., “I am good at resisting temptation”) are rated on a five-point Likert scale from 1 (“totally disagree”) to 5 (“totally agree”). SCS-KD showed a good internal consistency (α_HIQ_ = 0.85; α_HAA_ = 0.86).

### Statistical Analyses

**FIGURE 1:**
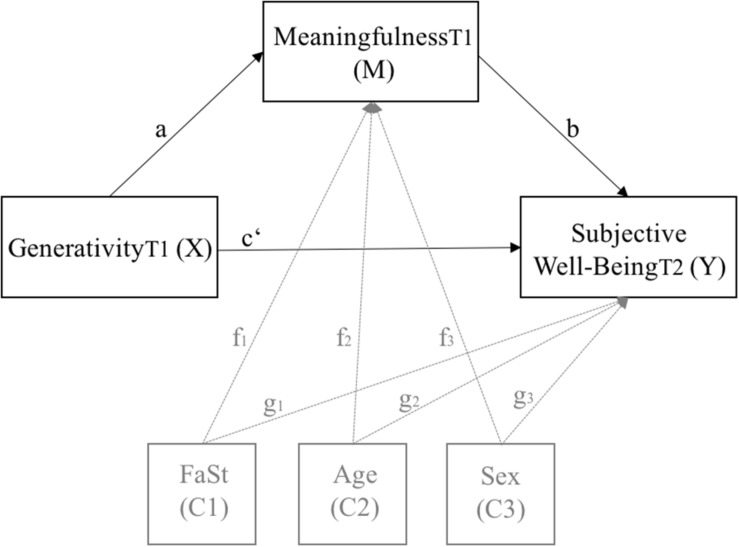
Statistical diagram of the simple mediation model with subjective well-being as dependent variable (*Y*), generativity as independent variable (*X*), and meaningfulness as mediator (*M*) while controlling for family status (C1), age (C2), and gender (C3).

**FIGURE 2:**
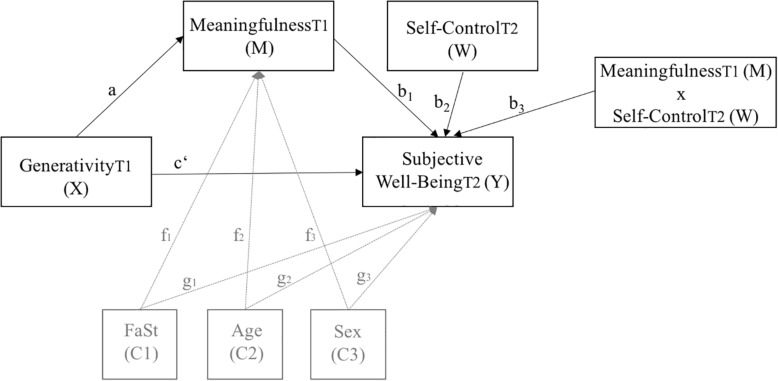
Statistical diagram of the moderated mediation model with subjective well-being as dependent variable (*Y*), generativity as independent variable (*X*), meaningfulness as mediator (*M*), and self-control as moderator (*W*) while controlling for family status (C1), age (C2), and gender (C3).

Statistical analyses were conducted using IBM Statistical Package for the Social Science (SPSS) version 24. First, we computed descriptive statistics, internal consistencies (Cronbach’s Alphas), inter-correlations, and mean-differences between t1 and t2. Correlation coefficients of *r* = 0.10−0.29 were interpreted as a weak effect, *r* = 0.30−0.49 as a moderate effect, and *r* ≥ 0.50 as a high effect (Cohen, 1988). Second, we employed multivariate analysis of covariance (MANCOVA) to test for group differences within the observed variables controlled for demographics (age, gender, family status). Third, we utilized PROCESS (Version 3.1), a macro for SPSS developed by Hayes (2013, 2018) to test (a) simple mediation analyses (model number 4) as well as (b) moderated mediation analyses (model number 14). We applied the simple mediation and moderated mediation model for the two gifted groups, respectively, with subjective well-being as dependent variable (Y), generativity as independent variable (X), meaningfulness as mediator (M), and self-control as moderator (W) (see Figures 1, 2). All analyses were controlled for demographics (family status [0 = no partner, 1 = partner] (C1), age (C2), and gender [0 = male, 1 = female] (C3)). We applied a bootstrap method suggested by Hayes (2018), 5000 resampling with replacement. This method has several advantages: It provides a reliable estimate of indirect effects; standard normality of the sampling distribution is not required; it has higher power and better Type I error control than other mediation analyses; it can be used for smaller sample sizes since it produces a distribution using the observed data to estimate statistical effects (Preacher and Hayes, 2008; Hayes, 2018). The significance of the models was assessed by examining the bootstrapped 95% confidence intervals, which should not include zero to meet the criterion of significance.

## Results

### Descriptive Statistics and Preliminary Analysis

Partial correlations (controlled for family status, age, and gender), 95% bias corrected and accelerated upper and lower confidence intervals, descriptive statistics (mean, standard deviation, and range), and scale reliabilities for all variables are shown in Table 1. Reported significance levels (*p*-values) and 95% bias corrected and accelerated upper and lower confidence intervals showed the same direction with regard to the significance of the reported associations with one exception - the association between meaningfulness and self-control among the HAA group. A statistical significance level of *p* < 0.05 suggested a significant weak association between the two variables while according to the 95% bias corrected and accelerated upper and lower confidence intervals, which had a zero included, the association would be interpreted as non-significant. Considering both significance measures, the following pattern of correlations was found in the HAA group: generativity showed a strong association with meaningfulness, but no significant association with subjective well-being or self-control. As expected, meaningfulness was moderately associated with subjective well-being and subjective well-being was strongly related to self-control. The pattern of correlations was slightly different in the HIQ group: generativity had a strong positive association with meaningfulness but no significant association with subjective well-being or self-control. Meaningfulness had a moderate association with subjective well-being and a weak association with self-control. Subjective well-being was moderately related with self-control. Cronbach’s alphas were good, ranging from α = 0.78 (for generativity among HIQ and meaningfulness in both groups) to α = 0.86 (for self-control among HAA).

**TABLE 1:** Partial correlations, descriptives, and reliabilities of generativity, meaningfulness, subjective well-being, and self-control among (a) intellectually gifted and (b) high academic achievers.

**Variables**	**1. Gen_T1_**		**2. MF_T1_**		**3. SWB_T2_**		**4. SC_T2_**	
	**HIQ**	**HAA**	**HIQ**	**HAA**	**HIQ**	**HAA**	**HIQ**	**HAA**
1. Gen_T__1_	(0.78)	(0.84)						
2. MF_T__1_	0.60^∗∗∗^ [0.44/0.74]	0.62^∗∗∗^ [0.39/0.78]	(0.78)	(0.78)				
3. SWB_T__2_	0.15 [−0.07/0.34]	0.24 [−0.06/0.48]	0.48^∗∗∗^ [0.32/0.60]	0.39^∗∗^ [0.11/0.59]	(0.82)	(0.80)		
4. SC_T__2_	0.10 [−0.13/0.32]	0.11 [−0.23/0.37]	0.29^∗∗^ [0.10/0.48]	0.28^∗^ [−0.10/0.58]	0.34^∗∗∗^ [0.17/0.48]	0.64^∗∗∗^ [0.39/0.80]	(0.85)	(0.86)
M	2.79	3.33	2.59	3.55	13.53	16.60	3.12	3.67
SD	1.00	1.05	1.06	1.02	4.41	4.21	0.65	0.60
Range	0–5	0–5	0–5	0–5	0–25	0–25	1–5	1–5

*Partial correlations were controlled for family status, age, and gender; HIQ, intellectually gifted group (*N* = 100); HAA, high academic achievers group (*N* = 52); Gen_*T*__1_, generativity at measurement time 1; MF_*T*__1_, meaningfulness at measurement time 1; SWB_*T*__2_, subjective well-being at measurement time 2; SC_*T*__2_, self-control at measurement time 2; Cronbach’s alphas are provided in parentheses on the diagonal; Pearson’s correlation coefficients: correlations of <0.10 = none, 0.10-0.29 = weak, 0.30-0.49 = moderate, ≥0.50 = high; ^∗^statistical significance level of *p* < 0.05; ^∗∗^statistical significance level of *p* < 0.01; ^∗∗∗^statistical significance level of *p* ≤ 0.001; Upper and lower levels of 95% bias corrected and accelerated confidence intervals are provided in squared brackets.*

*T*-tests for paired samples were conducted to test for individual stability of the variables (generativity, meaningfulness, and subjective well-being) between t1 and t2 (see Table 2). In the HIQ group, results revealed significant differences between t1 and t2 for meaningfulness (95%CI [−0.27, −0.03]; *d* = 0.25) and subjective well-being (95%CI [−2.32, −0.68]; *d* = 0.37); no differences were found for generativity. Both variables were higher at measurement time 2. Among the HAA, subjective well-being (95%CI [−2.28, −0.06]; *d* = 0.30) was significantly higher at t2, but no differences were found for generativity and meaningfulness. Inter-correlations of all variables were strong in both groups.

**TABLE 2:** Comparison of means and inter-correlations of generativity, meaningfulness, and subjective well-being at t1 and t2 among (a) intellectually gifted and (b) high academic achievers.

**(a) Intellectually gifted group (*N* = 100)**							
	**Means (SD)**						
**Variable**	***t*1**	***t*2**	**95% BaCI^#^**	***d***	**95%CI^+^**	**Inter-correlations^§^**	**95%CI^∗^**

Gen	2.79 (1.00)	2.90 (1.01)	[−0.23, 0.01]	0.18	[−0.09,0.46]	0.82	[0.75, 0.87]
MF	2.59 (1.06)	2.74 (1.01)	[−0.27, −0.03]	0.25	[−0.04,0.52]	0.83	[0.76, 0.88]
SWB	12.03 (5.24)	13.53 (4.41)	[−2.32, −0.68]	0.37	[−0.06,0.62]	0.65	[0.50, 0.76]

**(b) High academic achievers (*N* = 52)**							

	**Means (SD)**						
**Variable**	***t*1**	***t*2**	**95% BaCI^#^**	***d***	**95% CI^+^**	**Inter-correlations^§^**	**95% BaCI^∗^**

Gen	3.33 (1.05)	3.46 (1.05)	[−0.28, 0.03]	0.23	[−0.15,0.62]	0.86	[0.72, 0.94]
MF	3.55 (1.02)	3.58 (1.02)	[−0.19, 0.12]	0.05	[−0.33,0.44]	0.85	[0.75, 0.92]
SWB	15.42 (4.43)	16.60 (4.21)	[−2.28, −0.06]	0.30	[−0.10,0.68]	0.58	[0.31, 0.80]

*t1, measurement time 1; t2, measurement time 2; Gen, generativity; MF, meaningfulness; SWB, subjective well-being; #, upper and lower levels of 95% bias corrected and accelerated confidence intervals of mean differences; *d*, Cohen’s d; +, upper and lower levels of 95% bias corrected and accelerated confidence intervals of Cohen’s d; §, inter-correlations of t1 and t2; ^∗^, upper and lower levels of 95% bias corrected and accelerated confidence intervals of the inter-correlations of t1 and t2.*

A MANCOVA was utilized to test for group differences (HAA vs. HIQ) within the observed variables controlled for demographics (age, gender [0 = male, 1 = female], and family status [0 = no partner, 1 = partner]). A statistically significant general effect was found for the group variable [Wilks-Lambda = 0.867, *F*(4,144) = 5.539, *p* < 0.001, η^2^ = 0.133] and age [Wilks-Lambda = 0.927, *F*(4,144) = 2.828, *p* = 0.027, η^2^ = 0.073]. Older participants had the tendency to experience more meaningfulness in life (*F*(1,147) = 5.151, *p* = 0.025, η^2^ = 0.034) and subjective well-being (*F*(1,147) = 10.295, *p* = 0.002, η^2^ = 0.065). No statistically significant general effect was found for gender [Wilks-Lambda = 0.941, *F*(4,144) = 2.259, *p* = 0.066, η^2^ = 0.059] and family status [Wilks-Lambda = 0.944, *F*(4,144) = 2.153, *p* = 0.077, η^2^ = 0.056]. Intersubjective effects were significant for all psychological variables [generativity: *F*(1,147) = 5.141, *p* = 0.025, η^2^ = 0.034; meaningfulness: *F*(1,147) = 12.135, *p* = 0.001, η^2^ = 0.076; self-control: *F*(1,147) = 14.521, *p* < 0.001, η^2^ = 0.090] except for subjective well-being (*F*(1,147) = 1.725, *p* = 0.191, η^2^ = 0.012). HAA were thus established as being more generative, experiencing more meaningfulness and self-control. These findings confirm our hypothesis 2 and hypothesis 3.

### Simple Mediation Analyses

To test the hypothesis that generativity would lead to subjective well-being via a sense of meaning, a simple mediation analysis (PROCESS model 4; Hayes, 2018) was conducted for each of the two gifted groups. More specifically, we assumed that generativity would result in a sense of meaning and that a greater sense of meaning in life would increase subjective well-being among intellectually gifted adults (hypothesis 1a) as well as high academic achievers (hypothesis 1b). As can be seen in Table 3 and Figure 1, the proposed mediator (*M*), meaningfulness, was regressed on generativity (*X*) to produce path a. Subjective well-being (*Y*) was regressed on both meaningfulness (*M*) and generativity (*X*), which yielded path *b* and path *c*’, respectively. The simple mediation analyses were controlled for family status (C1; paths: *f*_1_, *g*_1_), age (C2; paths: *f*_2_, *g*_2_), and gender (C3; paths: *f*_3_, *g*_3_). As recommended by Hayes (2018), path coefficients were reported as unstandardized coefficients since standardized coefficients generally have no useful substantive interpretation. The two OLS regression equations representing the proposed model (see Figure 1) were

**TABLE 3:** Simple mediation model and unstandardized model coefficients among (a) intellectually gifted and (b) high academic achievers.

**(a) Intellectually gifted**						**Consequent**				
		**M (MEA)**					**Y (SWB)**			
**Antecedent**		**Coeff.**	**SE**	***p***	**95%CI**		**Coeff.**	**SE**	***p***	**95%CI**
X (GEN)	*a*	0.631	0.09	<0.001	0.46/0.80	c′	–0.911	0.47	0.056	−1.85/0.03
M (MEA)		–	–	–	–	*b*	2.447	0.45	<0.001	1.55/3.34
C1(FAST)	*f*_1_	0.451	0.19	0.019	0.07/0.83	g_1_	0.703	0.86	0.417	−1.01/2.41
C2 (AGE)	*f*_2_	0.018	0.01	0.072	−0.00/0.04	g_2_	0.005	0.05	0.914	−0.08/0.09
C3 (SEX)	*f*_3_	0.104	0.18	0.555	−0.25/0.45	g_3_	–2.671	0.78	0.001	−4.21/−1.13
Constant	*i*_M_	–0.349	0.54	0.516	−1.41/0.72	i_Y_	10.475	2.36	<0.001	5.79/15.16
		*R*^2^ = 0.404					*R*^2^ = 0.345			
		*F*(4,95) = 16.120, *p* < 0.001					*F*(5,96) = 9.896, *p* < 0.001			

**(b) High academic achievers**						**Consequent**				

X (GEN)	a	0.655	0.12	<0.001	0.41/0.90	*c*’	–0.054	0.68	0.937	−1.42/1.31
M (MEA)		–	–	–	–	b	1.500	0.64	0.024	0.20/2.80
C1(FAST)	*f*_1_	–0.389	0.41	0.345	−1.21/0.43	g_1_	2.009	1.82	0.274	−1.65/5.66
C2 (AGE)	*f*_2_	0.008	0.01	0.359	−0.01/0.03	g_2_	0.125	0.04	0.002	0.05/0.20
C3 (SEX)	*f*_3_	–0.016	0.27	0.953	−0.56/0.53	g_3_	0.918	1.19	0.448	−1.49/3.33
Constant	*i*_M_	1.277	0.65	0.056	−0.03/2.59	i_Y_	2.260	2.99	0.454	−3.76/8.28
		*R*^2^ = 0.435					*R*^2^ = 0.374			
		*F*(4,47) = 9.037, *p* < 0.001					*F*(5,46) = 5.499, *p* = 0.001			

*GEN, generativity; MEA, meaningfulness; FAST, family status; AGE, age; SEX, gender; SWB, subjective well-being; Coeff, unstandardized coefficient; SE, standard error; *p*, significance level; 95%CI, upper and lower levels of 95% bias corrected and accelerated confidence intervals.*

$$M=i_{M}+a\,X+f_{1}\,C_{1}+f_{2}\,C_{2}+f_{3}\,C_{3}+e_{M}$$

$$Y=i_{Y}+c^{\prime }\,X+b\,M+g_{1}\,C_{1}+g_{2}\,C_{2}+g_{3}\,C_{3}+e_{Y}$$

#### Simple Mediation Analysis for the HIQ Group

With regard to the coefficients of the simple mediation model in Table 3, the best fitting OLS regression model for the HIQ group was

$$\hat{M}=-0.349+0.631\,X+0.451\,C_{1}+0.018\,C_{2}+0.104\,C_{3}$$

$$\hat{Y}=10.475-0.911\,X+2.447\,M+0.703\,C_{1}+0.005\,C_{2}-2.671\,C_{3}$$

The results confirmed hypothesis 1a and showed that the indirect effect was statistically different from zero (indirect effect: ab = 1.544, bootstrap 95% CI [0.903, 2.230]; completely standardized indirect effect ab = 0.347, bootstrap 95% CI [0.202, 0.500]). Thus, two intellectually gifted individuals who differed by one unit in their generativity are estimated to differ by 1.544 units in their experienced subjective well-being as a result of the tendency for those being more generative to experience a higher sense of meaning in life, which in turn translated into higher subjective well-being. The direct effect of generativity on subjective well-being was not statistically different from zero (*t*(94) = −1.933, *p* = 0.056, 95% CI [−1.846, 0.025]). This mediation model explained 34.5% of the variance in subjective well-being in the HIQ group.

#### Simple Mediation Analysis for the HAA Group

Referring to the coefficients of the simple mediation model in Table 3, the best fitting OLS regression model for the HAA group was

$$\hat{M}=1.277+0.655\,X-0.389\,C_{1}+0.008\,C_{2}-0.016\,C_{3}$$

$$\hat{Y}=2.260-0.054\,X+1.500\,M+2.009\,C_{1}+0.125\,C_{2}+0.918\,C_{3}$$

The results confirmed hypothesis 1b and showed that the indirect effect was statistically different from zero (indirect effect: ab = 0.982, bootstrap 95% CI [0.124, 2.164]; completely standardized indirect effect ab = 0.244, bootstrap 95% CI [0.033, 0.522]). Thus, two academically high achievers who differed by one unit in their generativity were estimated to differ by 0.982 units in their experienced subjective well-being as a result of the tendency for those being more generative to experience a higher sense of meaning in life, which in turn translated into higher subjective well-being. The direct effect was not statistically different from zero (*t*(46) = −0.080, *p* = 0.937, 95% CI [−1.418, 1.310]). This mediation model explained 37.4% of the variance in subjective well-being in the HAA group.

### Moderated Mediation Analyses

To test whether the effect of meaningfulness on subjective well-being was contingent on trait self-control we combined the simple mediation model mentioned above with a moderation model and carried out a moderated mediation analysis (PROCESS model 14; Hayes, 2018) for both gifted groups, respectively. More specifically, we proposed that the positive effect of meaning in life on subjective well-being would be even stronger among intellectually gifted adults (hypothesis 4a) as well as high academic achievers (hypothesis 4b) high in trait self-control. As can be seen in Table 4 and Figure 2, meaningfulness (*M*) was regressed on generativity (*X*) to generate path a. Additionally, subjective well-being (*Y*) was regressed on generativity (*X*), which yielded path *c*’; on meaningfulness (*M*), which generated path b_1_; on trait self-control (*W*), which yielded path b_2_, as well as on an interaction between meaningfulness (*M*) and trait self-control (*W*), which produced b_3_. The moderated mediation analyses were controlled for family status (C1; paths: *f*_1_, *g*_1_), age (C2; paths: *f*_2_, *g*_2_), and gender (C3; paths: *f*_3_, *g*_3_). The two OLS regression equations representing this model (see Figure 2) were

$$M=i_{M}+a\,X+f_{1}\,C_{1}+f_{2}\,C_{2}+f_{3}\,C_{3}+e_{M}$$

$$Y=i_{Y}+c^{\prime }\,X+b_{1}\,M+b_{2}\,W+b_{3}\,M\,W+g_{1}\,C_{1}+g_{2}\,C_{2}+g_{3}\,C_{3}+e_{Y}$$

**TABLE 4:** Moderated mediation model and unstandardized model coefficients among (a) intellectually gifted and (b) high academic achievers.

**(a) Intellectually gifted group**						**Consequent**				
		**M (MEA)**					**Y (SWB)**			
**Antecedent**		**Coeff.**	**SE**	***p***	**95%CI**		**Coeff.**	**SE**	***p***	**95%CI**
X (GEN)	a	0.633	0.09	<0.001	0.46/0.80	c′	–1.140	0.47	0.017	−2.07/−0.21
M (MEA)		–	–	–	–	*b*_1_	–1.560	1.69	0.359	−4.92/1.80
W (SCO)		–	–	–	–	b_2_	–2.486	1.64	0.134	−5.75/0.78
M × W		–	–	–	–	b_3_	1.287	0.54	0.020	0.21/2.37
C1(FAST)	*f*_1_	0.126	0.05	0.014	0.03/0.23	g_1_	–0.076	0.23	0.736	−0.52/0.37
C2 (AGE)	*f*_2_	0.021	0.01	0.037	0.00/0.04	g_2_	0.011	0.04	0.780	−0.08/0.10
C3 (SEX)	*f*_3_	0.096	0.18	0.586	−0.25/0.44	g_3_	–2.296	0.76	0.003	−3.80/−0.79
Constant	*i*_M_	–0.497	0.55	0.368	−1.59/0.59	i_Y_	18.883	5.60	0.001	7.77/29.99
		*R*^2^ = 0.408					*R*^2^ = 0.404			
		*F*(4,95) = 16.370, *p* < 0.001					*F*(7,92) = 8.920, *p* < 0.001			

**(b) High academic achievers group**						**Consequent**				
X (GEN)	a	0.590	0.11	<0.001	0.37/0.81	*c*′	0.217	0.49	0.662	−0.78/1.21
M (MEA)		–	–	–	–	b_1_	2.769	1.85	0.142	−0.96/6.50
W (SCO)		–	–	–	–	b_2_	5.646	1.74	0.002	2.14/9.15
M × W		–	–	–	–	b_3_	–0.599	0.50	0.236	−1.60/0.41
C1(FAST)	*f*_1_	–0.087	0.12	0.478	−0.33/0.16	g_1_	–0.252	0.44	0.570	−1.14/0.64
C2 (AGE)	*f*_2_	0.010	0.01	0.240	−0.01/0.03	g_2_	0.108	0.03	0.001	0.05/0.17
C3 (SEX)	*f*_3_	0.105	0.26	0.684	−0.41/0.62	g_3_	0.487	0.91	0.594	−1.34/2.31
Constant	*i*_M_	1.248	0.68	0.074	−0.12/2.62	i_Y_	–12.297	6.53	0.066	−25.45/0.86
		*R*^2^ = 0.430					*R*^2^ = 0.618			
		*F*(4,47) = 8.864, *p* < 0.001					*F*(7,44) = 10.156, *p* < 0.001			

*GEN, generativity; MEA, meaningfulness; FAST, family status; AGE, age; SEX, gender; SWB, subjective well-being; Coeff, unstandardized coefficient; SE, standard error; *p*, significance level; 95%CI, upper and lower levels of 95% bias corrected and accelerated confidence intervals.*

#### Moderated Mediation Analysis for the HIQ Group

With regard to the coefficients of the moderated mediation model in Table 4, the best fitting OLS regression model for the HIQ group was

$$\hat{M}=-0.497+0.633\,X+0.126\,C_{1}+0.021\,C_{2}+0.096\,C_{3}$$

$$\hat{Y}=18.883-1.140\,X-1.560\,M-2.486\,W+1.287\,M\,W-0.076\,C_{1}+0.011\,C_{2}-2.296\,C_{3}$$

**FIGURE 3:**
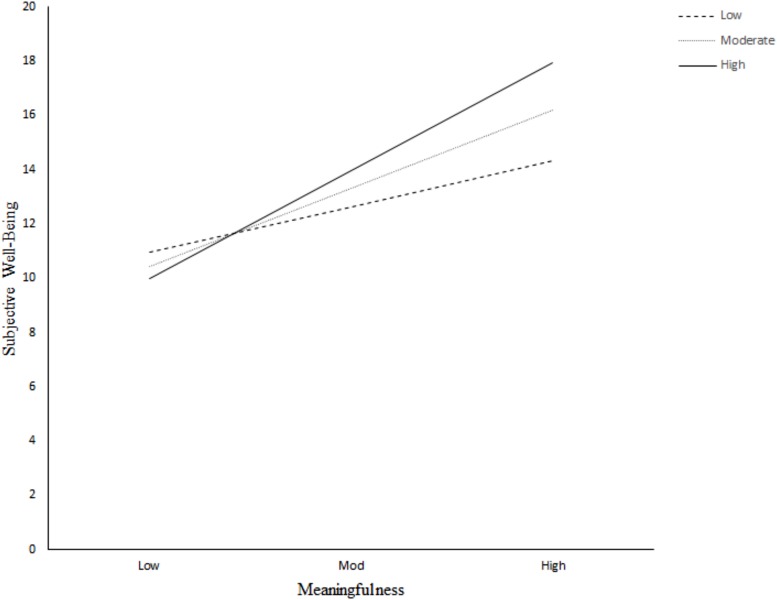
Conditional effect of meaningfulness on subjective well-being for low, moderate, and high levels of the trait self-control in the Intellectually Gifted Group.

These results confirmed hypothesis 4a and suggest that the more generativity was reported by the HIQ, the higher was their sense of meaning in life (*a* = 0.633, *p* < 0.001, 95% CI [0.464, 0.802]). Furthermore, the effect of meaningfulness on subjective well-being was contingent on an individual’s trait self-control, as evidenced by the statistically significant interaction between meaningfulness and trait self-control (*b*_3_ = 1.287, *p* = 0.020, 95% CI [0.207, 2.366]). In this model, *b*_1_ estimated the effect of meaningfulness on subjective well-being in gifted individuals measuring zero in trait self-control but scoring equally in generativity. This effect was not statistically significant from zero. The regression coefficient for trait self-control, *b*_2_, estimated the effect of trait self-control on subjective well-being among gifted adults reporting zero on meaningfulness. Again, this effect was not statistically significant from zero. This moderated mediation model explained 40.4% of the variance in subjective well-being in the HIQ group. Figure 3 illustrates the conditional effect of meaningfulness (*M*) on subjective well-being (*Y*) for low, moderate, and high levels (see Table 5) of trait self-control (*W*). As can be seen in the interaction graph, the higher meaningfulness, the higher subjective well-being among the HIQ. This relationship increased continuously with ascending self-control.

**TABLE 5:** Conditional indirect effects of meaningfulness (M) on subjective well-being (Y) at various levels of self-control (M) among (a) intellectually gifted and (b) high academic achievers.

			**Bias-corrected**	
**(a) Intellectually gifted group**			**95% BootCI**	
	**Effect**	**BootSE**	**Lower**	**Upper**
Self-control 16^th^ percentile	0.902	0.381	0.170	1.662
Self-control 50^th^ percentile	1.549	0.337	0.908	2.243
Self-control 84^th^ percentile	2.134	0.453	1.282	3.070

			**Bias-corrected**	
**(b) High academic achievers group**			**95% BootCI**	

Self-control 16^th^ percentile	0.574	0.446	−0.277	1.527
Self-control 50^th^ percentile	0.315	0.341	−0.513	0.864
Self-control 84^th^ percentile	0.097	0.433	−1.082	0.652

### Moderated Mediation Analysis for the HAA Group

Referring to the coefficients of the moderated mediation model in Table 4, the best fitting OLS regression model for the HAA group was

$$\hat{M}=1.248+0.590\,X-0.087\,C_{1}+0.010\,C_{2}+0.105\,C_{3}$$

$$\hat{Y}=-12.297+0.217\,X+2.769\,M+5.646\,W-0.599\,M\,W-0.252\,C_{1}+0.108\,C_{2}+0.487\,C_{3}$$

Similar to the HIQ group, higher generativity led to higher meaning in life among HAA (*a* = 0.590, *p* < 0.001, 95% CI [0.370, 0.810]). Contrary to our assumption, however, the effect of meaningfulness on subjective well-being was not conditional on trait self-control in this gifted group. The effect of meaningfulness on HAA’s subjective well-being measuring zero in trait self-control but equal in generativity was non-significant as well. Only the effect of trait self-control on subjective well-being while controlling for meaningfulness was significant (*b*_2_ = 5.646, *p* = 0.002, 95% CI [2.145, 9.147]). Thus, hypothesis 4b was rejected. Table 5 shows the conditional effect coefficients of meaningfulness (*M*) on subjective well-being (*Y*) for low, moderate, and high levels of trait self-control (*W*). As can be seen, no significant moderation effect of trait self-control was found in the HAA group.

## Discussion

The current study investigated the longitudinal effect of gifted individuals’ generative orientations on their subjective well-being via a sense of meaning. Furthermore, we examined how the effect of meaningfulness on subjective well-being varied across levels of self-control. These simple mediation and moderated mediation models were tested using longitudinal data from 152 gifted adults, separated in two groups: intellectually gifted (HIQ; *N* = 100) and high academic achievers (HAA; *N* = 52).

Recent findings (e.g., Wirthwein, 2010; Pollet and Schnell, 2017) have emphasized the need to differentiate between various gifted groups since gifted individuals are not a homogenous population. Our findings supported this approach to a large extent, as group differences occurred in all our measures apart from subjective well-being. HAA reported higher levels of generativity, meaning in life, and self-control than the HIQ group. Considering these findings, we encourage researchers to distinguish between different facets of giftedness such as, for example, high intellectual giftedness vs. high (academic) performance when studying adult gifted individuals.

In line with our hypotheses, findings demonstrated that the actualization of generative orientations in life enhanced both gifted groups’ sense of meaning and in further consequence their subjective well-being over time. Furthermore, the positive effect of a sense of meaning on subjective well-being was raised by self-control, at least in the HIQ group. Thus, an interaction of meaningfulness and a certain level of intrinsic willpower to subdue one’s inner responses, emotions as well as undesired behaviors appears to be of particular significance for their happiness. Contrary to our hypothesis, self-control did not enhance the positive association between meaningfulness and subjective well-being among HAA. However, results suggested, as proposed, that their subjective well-being was strongly related to self-control. Considering their high achievement and perfect fit with educational standards (from early school stages on) (e.g., Vötter and Schnell, 2019b), these results are in line with recent findings which suggest a positive association between self-control and academic success (e.g., Tangney et al., 2004; Duckworth and Seligman, 2005) as well as happiness (e.g., Cheung et al., 2014).

Since our findings showed a positive effect of generativity on meaning in life and, in further consequence, on higher subjective well-being over time, by implication, this source of meaning might be especially important for those gifted individuals who are suffering from a crisis of meaning (e.g., Vötter and Schnell, 2019b). Accordingly, future research is needed to further examine if caring for future generations, or creating some kind of legacy might help when suffering from an existential crisis. Results of a recent interventional study by Gruenewald et al. (2016) showed striking evidence that self-perception as being high in generativity can be enhanced through certain generative tasks (e.g., voluntary tutoring of school children). Giving these promising findings, trainings designed to alter gifted individuals’ sense of contributing to others’ lives may in turn lead to a feeling of significance and a sense of belonging. In further consequence, this may help them to live a more meaningful life and to find a way out of existential crises. As mentioned before, there are multiple ways to express generativity. Volunteering—giving one’s time, knowledge, or abilities to organizations and individuals without the expectation of payment—as well as pro-social participation might be a significant source of generativity (e.g., Schnell and Hoof, 2012) among the gifted. Volunteering is often associated with being part of a community; it might thus strengthen a sense of fitting-in and connectedness, also among gifted individuals. Also passing on knowledge and skills to others in form of teaching or mentoring is a way of living generativity (e.g., Rossi, 2001; Gruenewald et al., 2016). Even donating money to a charitable fund might lead to a sense of generativity (e.g., van Tilburg and Igou, 2013) although, in this case, the significance of one’s action is often not visible.

### Strengths, Limitations, and Future Research

The longitudinal design of the study is surely one of its strengths. The design made it possible to analyze the stability of the observed variables as well as the prediction of associations between the variables with greater certainty than would have been possible in a cross-sectional design. Another strength of our study is the diversity of our two gifted groups. The results can give us valuable insights into differential developmental needs of intellectually gifted and high achieving adults toward a happy life. Notwithstanding its strengths, we would like to note some important limitations of the current study that should be addressed in future research. First, found relationships might not be causal, but influenced by further, non-assessed constructs. Second, our samples comprised of two preselected gifted groups: members of the high IQ society Mensa and academic achievers who were honored with a *promotio sub auspiciis praesidentis rei publicae.* Furthermore, our participants were mostly Austrians and Germans. Therefore, generalizability of our results to unselected gifted individuals, or to other cultures, cannot be claimed. Baudson (2016) noted that particularly in Germany a negative stereotype about giftedness might predominate. Thus, the literature would benefit from future longitudinal studies that examine meaningfulness and subjective well-being related issues among Mensa members and other (non-preselected) HIQs with various nationalities to establish if our results are valid on an international level. Furthermore, an ideal design would include a control group of individuals with average IQ to get more comparable results. Due to the complexity of the longitudinal design, this could not be realized in the current study. Third, since the data was collected via an online survey, the link to the survey could have been shared with individuals outside of our target groups. However, we did control for this possibility by asking the yes/no-questions “Are you a member of the high IQ society Mensa?” and “Have you been honored with a *promotio sub auspiciis praesidentis rei publicae* award?” Nevertheless, we were dependent on participants’ honesty. Fourth, 29% of the participants in the HAA group were female. This rather low proportion of females in the sample could be assumed to lead to gender biased results. To control for this bias, we included gender as well as the other socio-demographics as covariates in all analyses. Moreover, the gender ratio in our HAA sample (29% female) matches exactly the gender ratio of the total population of those honored with the *promotio sub auspiciis praesidentis rei publicae* (*N* = 1042) and is therefore representative for this specific gifted group. Fifth, the sample size of the HAA group is rather small (*N* = 52). However, considering (a) the strict requirements for being honored with a *promotio sub auspiciis praesidentis rei publicae* award plus the fact that since 1952 only slightly over 1000 individuals have received this honor, (b) that recent findings (Wirthwein, 2010; Pollet and Schnell, 2017) emphasized the need to differentiate between gifted groups, and (c) that our data is longitudinal, we argue that examining two gifted groups—in spite of the rather small sample size—can be seen as a strength of this study rather than a limitation. Furthermore, the aforementioned power analysis indicated sufficient power to detect medium effects. To account for the small sample size, we used bootstrapping in all simple mediation and moderated mediation analyses. This method is recommended for smaller sample sizes since it uses observed data to produce a distribution for the estimation of statistical effects (Preacher and Hayes, 2008; Hayes, 2018). One could argue that this rather small sample size limits the range of analyses which can be conducted. However, the chosen conditional process analyses are deemed appropriate for the proposed context, since, according to Hayes (2018), maximum likelihood-based structural equation modeling (SEM) is not necessarily better or more appropriate than the usage of mediation, moderations and conditional process analyses with PROCESS. Sixth, the longitudinal data of the current study is based on self-reports, thus posing the risk of selective recollection of data as well as over- or under-reporting. However, the assessed scales have been verified as reliable and valid measures throughout numerous studies (e.g., Brähler et al., 2007; Schnell and Becker, 2007; Bertrams and Dickhäuser, 2009).

## Conclusion

Results highlight the positive effect of generativity on gifted individuals’ sense of meaning and happiness over time. Particularly among intellectually gifted individuals—who have been found to be at a higher risk of existential crisis (Vötter and Schnell, 2019b)—this effect is conditional on trait self-control. Thus, an intrinsic willpower to subdue inner responses, emotions as well as undesired behaviors could strengthen the positive effect between meaning in life and subjective well-being. While high cognitive abilities can be seen as a gift that enables empowerment, they also seem to be a predictor of disharmonious socioemotional development, as suggested by several scholars (e.g., Karprinski et al., 2018; Vötter and Schnell, 2019a, b). Crucial questions about underlying mechanisms and personality traits remain: Why do some intellectually gifted individuals differ from others, and how can their psychological and mental health be supported? Further research is necessary to understand to which degree differences in gifted adults’ life-worlds are caused by their personality, by negative experiences in family or educational settings (e.g., Pollet and Schnell, 2017; Vötter and Schnell, 2019b), or by interactions thereof.

## Data Availability

The datasets generated for this study are available on request to the corresponding author.

## Ethics Statement

This study was carried out in accordance with the Declaration of Helsinki. Participation was entirely voluntary, after a free and informed consent. All participants declared their consent by signing up for participation of their own accord, after receiving a written invitation and study information. All participants were made aware that they could withdraw from the study at any time. In accordance with legislation and guidelines in Austria and the University of Innsbruck, formal approval by an ethics advisory board was not required for this type of study.

## Author Contributions

Both authors contributed equally to the design of the study. BV executed the study, conducted the data analyses, and wrote the manuscript. TS advised on the execution and analyses of the study, and collaborated in the editing of the manuscript.

## Conflict of Interest Statement

The authors declare that the research was conducted in the absence of any commercial or financial relationships that could be construed as a potential conflict of interest.
